# Reduced CD4^+^T Cell CXCR3 Expression in Patients With Allergic Rhinitis

**DOI:** 10.3389/fimmu.2020.581180

**Published:** 2020-11-03

**Authors:** Xiaofeng Yu, Meng Wang, Zhiwei Cao

**Affiliations:** Department of Otolaryngology Head and Neck Surgery, Shengjing Hospital of China Medical University, Shenyang, China

**Keywords:** CD4^+^ T Cell, CXCR3, allergic rhinitis, T cell subset, cytokines

## Abstract

While T cells are considered to play a primary role in IgE-mediated atopic diseases, little is known about the systemic variations of T cell subsets from patients with allergic rhinitis (AR). To elucidate the characteristics of peripheral T cells, we analyzed natural killer, B cell, and T cell populations, performed T cell subset construction, and assessed chemokine receptor and associated serum cytokine expression in 25 AR patients and 20 healthy controls. Our results revealed increased levels of CD4^+^T cells, serum interleukin (IL)-10, IL-6, and interferon (IFN)-γ, and reduced Th1 and Th17 subsets, identified by their chemokine receptors, in AR patients. These results suggest a systemic activation of T cell responses in AR. We further demonstrated that AR patients exhibit significantly reduced CD4^+^T cell CXCR3 expression, especially in patients with moderate-severe disease severity, demonstrating that CXCR3 is a potential key molecule that hinders the Th1/Th2 balance in AR pathology. Overall, systemic T cell activation occurred in AR patients and CXCR3 dramatically decreased in CD4^+^T cells, which may ultimately be used as a potential disease and/or therapeutic target.

## Introduction

Allergic rhinitis (AR) is a disease characterized by IgE-mediated allergic inflammation of the nasal mucosa after allergen exposure, associated with nasal symptoms including rhinorrhea, sneezing, nasal obstruction, and nasal itchiness. In AR, allergen exposure triggers allergic responses, and sensitization involves antigen-presenting cells, T and B lymphocytes, and results in the generation of allergen-specific T cells and allergen-specific IgE antibodies. Upon re-exposure to allergens, crosslinking of IgE on mast cells results in the release of mediators of hypersensitivity, such as histamine and immediate nasal symptoms. Within hours, there is an infiltration of inflammatory cells, notably Th2 T lymphocytes, eosinophils, and basophils, into nasal mucosal tissue that results in the late-phase allergic response.

Several studies have demonstrated obvious variations of peripheral T cell subsets in AR patients, such as elevated peripheral Th2 cells (1), Th17 cells (2), follicular helper T cells (3), CD161^+^ T cells (4), and reduced CD4^+^ terminally differentiated effector memory T cells (5). However, the whole profile of peripheral lymphocytes, such as natural killer (NK), B, and T cell subsets, has not been fully described in AR patients with different disease severity, which will ultimately provide a better understanding of the pathophysiology of AR.

The interaction between chemokines and their receptors is an important step in the control of a variety of lymphocyte activities, including migration into sites of inflammation and induction and enhancement of cytokine responses under polarizing conditions. Recent studies have shown that human Th1, Th2, and Th17 clones display distinct patterns of chemokine receptor expression. Th1 clones preferentially express CXCR3, Th2 clones express CCR4, and many Th17 clones express CCR6 (6, 7); this can be used in identifying peripheral T cell subsets without polarization.

In this study, we investigated differences in peripheral lymphocyte subsets, based on their chemokine receptor expression and cytokines, in AR patients and heathy controls. We hypothesized that chemokine receptor expression on T cell subsets would change during allergic inflammatory reactions, and assessed chemokine receptor expression on T cell subsets to identify a possible relationship between expression level and AR disease severity.

## Materials and Methods

### Subjects

Twenty-five patients with AR and 31 healthy controls (HCs, peripheral blood mononuclear cells were collected from 20 HCs) were recruited from October 2019 to February 2020 from the Otorhinolaryngology clinic in Shengjing Hospital affiliated to China Medical University (CMU). All the 25 patients are primary for our clinic and claimed no immunotherapy. The severity of AR was diagnosed by a physician, based on the presence of clinical symptoms, nasal endoscopic examination, and patient allergy history. The classification of mild and moderate-severe AR was based on the Allergic Rhinitis and its Impact on Asthma (ARIA) guidelines (8).

All AR patients were divided into two groups according to their disease severity, namely mild patients (score 1) and moderate to severe patients (scores 2 and 3). Ethics approval was obtained from the Ethics Committee of Shengjing hospital affiliated to CMU, and written informed consent for participation in the study was obtained from all study participants.

### Blood Sample Processing

Five milliliters of peripheral blood was collected from each study participant with vacutainer tubes containing ethylenediaminetetraacetic acid (EDTA; Becton Dickinson, Plymouth, UK). Peripheral blood mononuclear cells (PBMCs) were obtained by Ficoll–Hypaque density gradient centrifugation, before being cryopreserved in fetal calf serum supplemented with 10% dimethyl sulphoxide, and stored in liquid nitrogen within 48 h of collection. Levels of total IgE in the plasma were measured in AR patients using ImmunoCAP 100 (Phadia, Uppsala, Sweden).

### Determination of Lymphocyte Subset Counts

Lymphocyte subset count (T, B, NK, CD4, and CD8) percentages and absolute numbers were determined with a BD FACSCanto™ II (Becton Dickinson, USA) flow cytometer, using a six-color direct immunofluorescence reagent (BD Multitest IMK kit and BD Multitest 6-color TBNK, Becton Dickinson, USA) with trucount tubes (Becton Dickinson, USA).

### Detection of CD4^+^T Cell Subset and Receptor Expression

**Figure 1:**
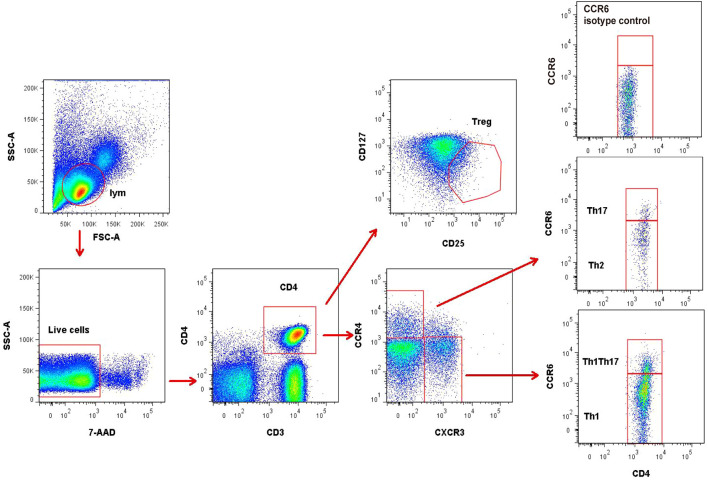
Representative flow cytometry gating strategy for identification of CD4+T subsets. Treg (CD25^+^CD127^low/-^), Th1 (CXCR3^+^CCR4^−^CCR6^−^), Th2 (CXCR3^−^CCR4^+^CCR6^−^), and Th17 (CXCR3^−^CCR4^+^CCR6^+^ and CXCR3^+^CCR4^−^CCR6^+^) in peripheral blood mononuclear cells. The data were obtained from 1 healthy control subject.

Regulatory T cells (Treg) were defined as CD4^+^CD25^+^CD127^low/-^ cells. Other CD4^+^T subsets were identified by differential expression of CCR4, CXCR3, and CCR6 as previously reported (7, 9): CXCR3^+^CCR4^−^CCR6^− ^(Th1), CXCR3^−^CCR4^+^CCR6^−^ (Th2), CXCR3^−^CCR4^+^CCR6^+^(Th17), and CXCR3^+^CCR4^−^CCR6^+^ (Th1Th17) (**Figure 1**). In addition, Th17 cells were defined as the summation of CXCR3^−^CCR4^+^CCR6^+^ (Th17) and CXCR3^+^CCR4^−^CCR6^+^(Th1Th17).

The fluorochrome-conjugated antibodies used for polychromatic flow cytometry analysis were CD3-BV510 (Biolegend, USA, catalog no. 317332), CD4-APC-Cy7 (Biolegend, USA, catalog no. 317418), CCR4-APC (Biolegend, USA, catalog no. 359404), CXCR3-PE (Biolegend, USA, catalog no. 353706), CCR6-PE-Cy7 (Biolegend, USA, catalog no. 353418), CD25-BV421 (Biolegend, USA, catalog no. 302630), CD127-FITC (Biolegend, USA, catalog no. 351312), PE-Cy7-CCR7 (Biolegend, USA, catalog no. 353226) and CD62L-FITC (Biolegend, USA, catalog no. 304804). A viability dye, 7-AAD (Becton Dickinson, USA, catalog no. 559925), was used to exclude dead cells. Isotype antibodies were also used as negative controls for every detection to set proper gating for the receptor expression. Cells were analyzed by fluorescence-activated cell sorting (FACS), using the BD LSRII cytometer and FlowJo software.

### Measurement of Serum Cytokines and Chemokines

Serum was collected from AR patients and HCs, separated by centrifugation (2000 × *g* for 10 min), and stored in 500 µL aliquots at −80°C until analysis. Serum concentrations of IL-4, IL-2, IP-10, IL-6, TNF-α, MCP-1, IL-17, IL-10, IFN-γ, IL-12, and IL-8 were measured with the LEGENDplex human essential immune response panel (Biolegend, USA, catalog no. 740929), as per the manufacturer's instructions. Briefly, 50 μL of serum was incubated with antibody-coated capture beads for 2h at room temperature. After washing the beads, 25 μL of detection antibodies was added, and incubated with the beads for 1h at room temperature. Next, 25 µL of SA-PE was added directly to each well, and incubated for 30min at room temperature. After washing away unbound SA-PE, the beads were resuspended in sheath fluid for 5 min. Finally, samples were assessed on the BD FACSCanto II (Becton Dickinson, USA), and analyzed with LEGENDplex 8.0 software (Biolegend, USA).

### Statistical Analysis

Statistical calculations were performed using SPSS 17.0 software. Categorical data were described and analyzed by frequency and chi-square (χ^2^) tests. For parametric comparisons, two-tailed Mann-Whitney U or paired Wilcoxon tests were used to assess differences among groups. The Spearman's rank correlation test was used to measure correlations between variables. Unless otherwise stated, p values < 0.05 were considered statistically significant.

## Results

### Characteristics of Subjects

Twenty HCs, as well as six mild and nineteen moderate-severe AR patients were recruited in this study. **Table 1** summarizes the demographic and clinical characteristics of these subjects.

**Table 1:** Characteristics of AR patients and HCs.

Characteristic	HCs	AR patients(Mild)	AR patients (Moderate-Severe)	*p*-value
n	31	6	19	NA
Male, no. (%)	9 (29)	4 (66)	7 (37)	0.496
Age, mean (range), years	33 (25-61)	29 (19-42)	31 (23-63)	0.566
Mean total plasma IgE (IU/mL) ± SD		66.85 (89.85)	245.41 (356.28)	0.121
Symptoms classification (%)				
Intermittent		1	2	
Persistent		5	17	
**Nasal allergen challenge (positive %)**				
dust mite		52.63	47.37	
cat/dog dander		16.67	5.26	
cockroach		0	10.53	
Alternaria		16.67	5.26	
ragweed/grass		16.67	10.53	

HCs, healthy controls; AR, allergic rhinitis; NA, not applicable.

No significant difference in age and gender was found between HCs, mild AR patients, and moderate-severe AR patients. There was no significant difference in total plasma IgE levels between mild and moderate-severe AR patients.

### AR Patients Present Less Th1 and Th17 Cells Based on Cell Surface Expression of Chemokine Receptors

**Figure 2:**
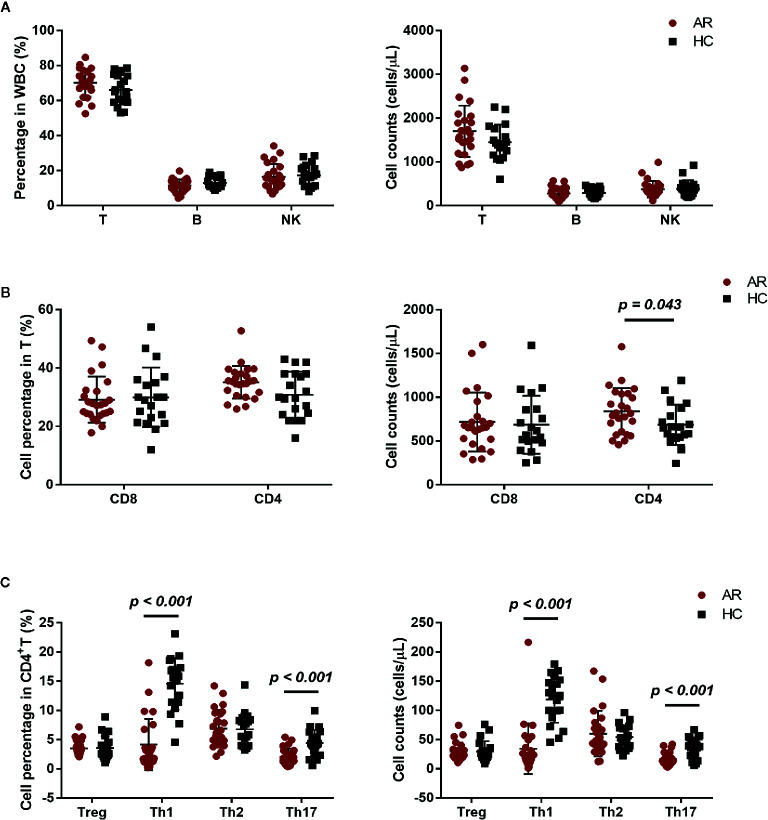
Cell subset expression in AR patients (n = 25) and HCs (n = 20). **(A)** Percentages and absolute counts of lymphocyte subsets: T cells (CD3^+^), B cells (CD3^-^CD19^+^), and NK cells (CD3^-^CD16/56^+^). **(B)** Percentages and absolute counts of T cell subsets: CD8 cells (CD3^+^CD8^+^) and CD4 cells (CD3^+^CD4^+^). **(C)** Percentages and absolute counts of CD4+ T cell subsets: Treg (CD25^+^CD127^low/-^), Th1 (CXCR3^+^CCR4^−^CCR6^−^), Th2 (CXCR3^−^CCR4^+^CCR6^−^), and Th17 (CXCR3^−^CCR4^+^CCR6^+^ and CXCR3^+^CCR4^−^CCR6^+^). Horizontal lines indicate mean ± SD. P-values were calculated with the two-tailed Mann-Whitney U test.

We investigated the percentage and absolute count of peripheral lymphocyte subsets in AR patients and HCs. There was no significant difference in T, B, and NK cell counts between AR patients and HCs, but a mild increased trend of T cells was found in AR patients (**Figure 2A**). We next determined T cell subset percentages and absolute counts and found that AR patients had more absolute CD4^+^T cell counts than HCs (**Figure 2B**).

**Figure 3:**
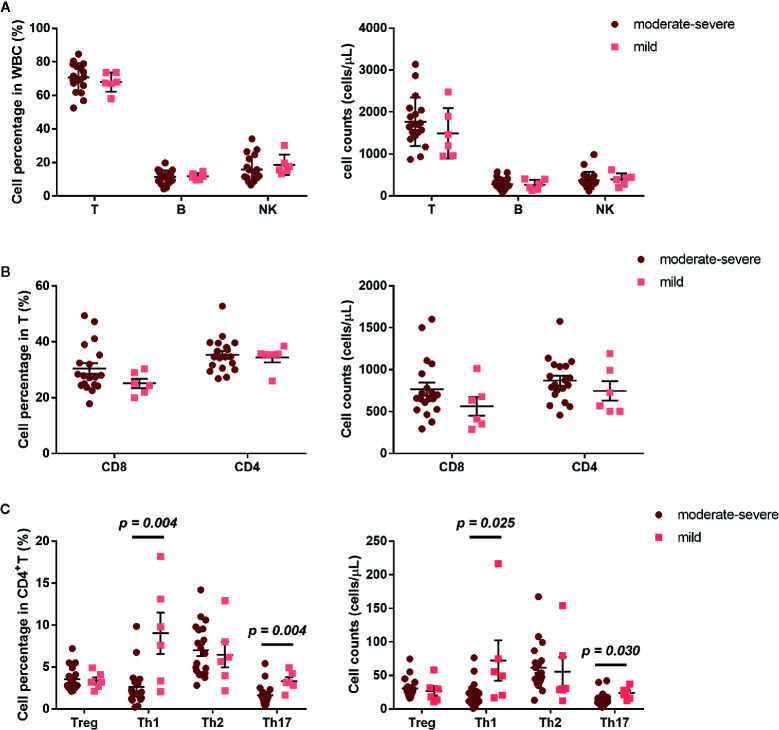
Cell subset expression in mild (n = 6) and moderate-severe (n = 19) AR patients. **(A)** Percentages and absolute counts of lymphocyte subsets: T cells (CD3+), B cells (CD3^-^CD19^+^), and NK cells (CD3^-^CD16/56^+^). **(B)** Percentages and absolute counts of T cell subsets: CD8 cells (CD3^+^CD8^+^) and CD4 cells (CD3^+^CD4^+^). **(C)** Percentages and absolute counts of CD4^+^ T cell subsets: Treg (CD25^+^CD127^low/-^), Th1 (CXCR3^+^CCR4^−^CCR6^−^), Th2 (CXCR3^−^CCR4^+^CCR6^−^), and Th17 (CXCR3^−^CCR4^+^CCR6^+^ and CXCR3^+^CCR4^−^CCR6^+^). Horizontal lines indicate mean ± SD. P-values were calculated with the two-tailed Mann-Whitney U test.

The development of AR is associated with an imbalance between Th1/Th2 (10). We further investigated the frequency of Treg, Th1, Th2, and Th17, defined by their surface receptor expression in AR patients and HCs. AR patients showed significantly less Th1 and Th17 cells, both in percentage and absolute count (**Figure 2C**). In addition, we also compared NK, B, T, CD4, CD8, Treg, Th1, Th2, and Th17 cells between AR patients with mild and moderate-severe severity. Moderate-severe AR patients showed a significant reduction in Th1 and Th17 cells compared to mild AR patients (**Figure 3**). Collectively, in addition to increased peripheral CD4^+^T cell counts, AR patients exhibit an obvious downregulation of Th1 and Th17, especially patients with moderate-severe disease severity.

### T Cell Polarization Associated Cytokine Profile in AR Patients

Our data suggest an obvious downregulation of Th1 and Th17 cells in AR patients, especially in those with moderate-severe disease severity. However, several studies have reported increased Th1 and Th17 cytokines, such as INF-γ, IL-17, and IL-6, in AR patient sera (2). Thus, we analyzed serum Th1/Th2/Th17 cytokines in AR patients and HCs. Contrary to the decreased Th1 and Th17 subset defined by surface markers, we found that AR patients in this study had increased levels of IFN-γ, IL-10 and IL-6, which suggests an upregulation of both Th1 and Th17 cytokines (**Table 2**).

**Table 2:** Characteristics of serum cytokines.

Mean (SD) pg/mL	AR patients (n = 25)	HCs (n = 31)	*p*-value
**IL-2**	2.56 (0.80)	2.50 (0.38)	0.532
**IL-12**	3.21 (2.40)	2.51 (2.29)	0.451
**IFN-γ**	8.84 (6.83)	5.68 (2.60)	0.049*
**IP-10**	166.56 (120.80)	154.18 (50.13)	0.252
**IL-4**	8.41 (6.29)	7.39 (4.44)	0.839
**IL-10**	3.30 (4.95)	1.42 (0.48)	0.003*
**IL-6**	18.85 (15.06)	9.27 (8.64)	0.009*
**IL-17**	1.96 (1.33)	1.57 (0.56)	0.382
**TNF-α**	2.66 (1.37)	2.62 (0.53)	0.168
**MCP-1**	179.34 (83.70)	161.81 (80.04)	0.349
**IL-8**	6.96 (6.49)	12.70 (9.60)	0.007*

IL, interleukin; IP, interferon-γ-inducible protein 10; TNF-α, tumor necrosis factor alpha; MCP-1, monocyte chemoattractant protein-1; IFN-γ, interferon gamma. *difference statistically significant at p < 0.05.

### Decreased Surface CXCR3, Not Th1 and Th17, Is the Predominant Feature of CD4^+^T Cells in AR Patients

**Figure 4:**
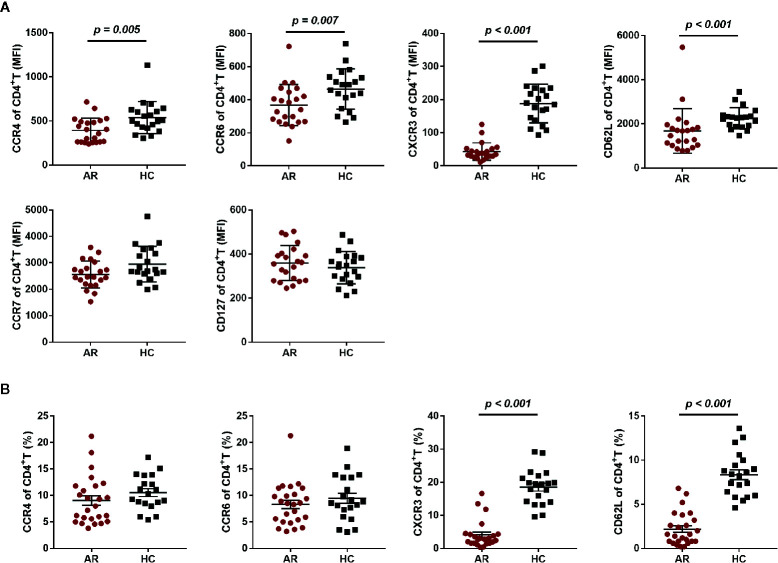
Chemokine and cytokine receptor expression on CD4+T cells in AR patients and HCs. **(A)** The mean fluorescence intensity (MFI) of CCR4, CCR6, CXCR3, CD62L, CCR7, and CD127 on CD4^+^T cells is shown for AR patients (n = 25) and HCs (n = 20). **(B)** The frequency of CCR4^+^, CCR6^+^, CXCR3^+^, and CD62L^+^ cells in CD4^+^T cells is shown for AR patients (n = 25) and HCs (n = 20). Horizontal lines indicate mean ± SD. P-values were calculated with the two-tailed Mann-Whitney U test.

Given the increased serum cytokines of Th1 and Th17, we speculated that the reduced Th1 and Th17 subsets observed in this study may be attributed to expression variations in the associated chemokine receptors. Thus, we investigated chemokine and cytokine receptors on CD4^+^T cells, such as CXCR3, CCR4, and CCR6. We first compared the mean fluorescence intensity (MFI) of CCR7, CCR4, CCR6, CXCR3, CD127, and CD62L on CD4+T cells between AR patients and HCs, and found a significantly reduced expression of CCR4, CCR6, CXCR3, and CD62L on CD4^+^T cells (**Figure 4A**). We also analyzed CCR4^+^, CCR6^+^, CXCR3^+^, and CD62L^+^ cell frequencies in CD4^+^T cells, and found that the frequency of CXCR3^+^CD4^+^T cells and CD62L^+^CD4^+^T cells was significantly decreased in AR patients (**Figure 4B**).

**Figure 5:**
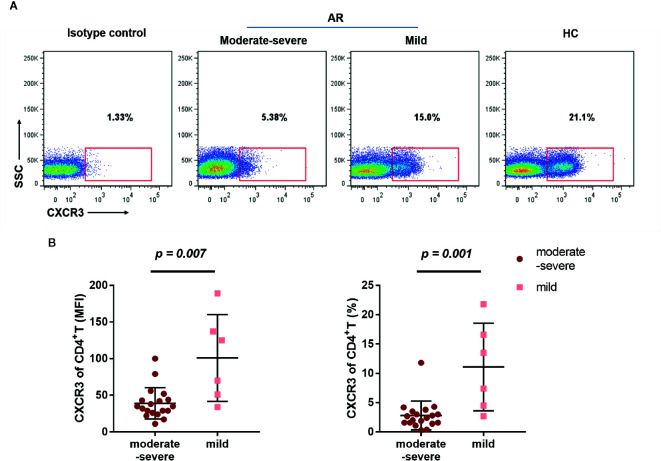
Downregulated CXCX3 expression on CD4^+^T cells in AR patients. **(A)** A representative flow cytometry plot showing the different percentages of CXCR3 on CD4^+^T cells in HC, mild, and moderate-severe AR patients. **(B)** Comparison of MFI and percentages of CXCR3 on CD4^+^T cells (CD4^+^CXCR3^+^% of CD3^+^T cells) between mild (n = 6) and moderate-severe (n = 19) AR patients. Horizontal lines indicate mean ± SD. P-values were calculated with the two-tailed Mann-Whitney U test.

Next, we compared the differential expression markers between mild and moderate-severe AR patients, and found that CXCR3 was significantly downregulated by both MFI and frequency in AR patients with moderate-severe disease severity (**Figure 5**). Collectively, our data indicated that decreased surface CXCR3, not Th1 and Th17, is the predominant feature of AR patient CD4^+^T cells.

## Discussion

An allergic response in AR patients begins with the deposition of allergens into the nasal mucous membrane. These allergens are taken up and processed by antigen-presenting cells and presented to T cells, which will become activated. Th2 cells are the main T cell subsets involved in allergic diseases, which release effector cytokines, such as IL-4, IL-10, and IL-13, that will interact with B cells for the synthesis of allergen-specific IgE. IgE binds to a high affinity receptor on the surface of mast cells, leading to the release of mediators that trigger the nasal symptoms of rhinorrhea, sneezing, nasal itchiness, and blockage (11, 12). In the present study, we investigated differences between peripheral lymphocytes, T cell subsets, and serum cytokines in AR patients and HCs. We found that reduced peripheral blood Th1 and Th17 subsets markedly separated AR patients from HCs, as well as AR patients with moderate-severe or mild symptoms. However, in serum cytokine detection, Th1 and Th17 cytokines, such as interferon (IFN)-γ and IL-17, did not show a reduction similar to the subset variations in AR patients. Indeed, serum IL-6 and IFN-γ levels were significantly increased in AR subjects. Given that T cell subsets were distinguished by chemokine receptors in this study, we further investigated the potential effects of allergic responses on T cell chemokine receptor expression, such as CXCR3, CCR4, and CCR6. Significantly reduced CXCR3 expression on CD4^+^T cells was observed in AR patients, especially in those with moderate-severe symptoms. Thus, decreased surface CXCR3, not Th1 and Th17, is the predominant feature of CD4^+^T cells in AR patients. Through investigation of chemokine receptor expression in AR patients, we identified a significant reduction in CXCR3 expression on peripheral CD4^+^T cells, which was also associated with the disease severity of AR.

In this study, we found increased CD4^+^T cell counts and serum IL-10 in AR patients, which suggests a systemic activation of T responses. Several studies using peripheral blood samples of intermittent AR patients showed no significant difference in total CD4^+^ T cells between AR patients and HCs (1). However, most of the subjects in this study suffered persistent symptoms of AR, which is more likely to induce a systemic activation and expansion of CD4^+^T cells. For the serum cytokines, the nasal symptoms of AR are regulated by the local production and release of several cytokines, such as IL-1, TNF-α, and IL-6 (13). However, it is not only the local condition of the nasal mucosal tissue that affects cytokine levels. In agreement with previous studies (13–15), we found an upregulation of serum Th2 cytokine IL-10, Th1 cytokine IFN-γ, and Th17 cytokine IL-6 in AR patients. Viral infection also produced increased levels of IL-6 and IFN-γ, however, AR patients did not show elevated levels of other cytokines associated with viral infection, such as IP-10, IL-8, MCP-1, and TNF-α (16, 17). Collectively, the systemic increase in inflammatory responses triggered by AR is different from those induced by viral infection, and should be treated differently.

Accumulating evidence indicates a crucial role of Th1/Th2 cytokine imbalance in AR patients, who shift to more intensively Th2-dominated responses upon re-exposure to allergens, as opposed to non-allergic individuals who display a greater Th1 response (18, 19). In addition to the abundant cell activation and cytokine production in local tissue, T cell nasal migration also plays a primary role in Th2 polarization in AR. T cell migration is finely regulated by a family of small proteins called chemokines. During the process, T cells use surface chemokine receptors to selectively bind to certain chemokines and trigger transendothelial migration in the local tissue. Therefore, chemokine receptors can function as phenotypic markers for certain cell subsets. CCR4 and CCR6 have been suggested as Th2 and Th17 markers, respectively, whereas CXCR3 has a Th1 phenotype (20). We found that the MFI of CCR4 expressed on CD4^+^T cells was reduced in AR patients, however, the frequency of CCR4 ^+^CD4^+^T cells in AR is similar to that in HCs. This may be explained by elevated expression of its ligand, CCL22, was previously found in AR subjects (20). CXCR3 and its ligand IP-10 are increased in autoimmune diseases, such as rheumatoid arthritis and multiple sclerosis (21, 22). Here, we found that AR patients possess a significantly reduced CXCR3 expression in CD4^+^T cells, especially in patients with moderate-severe severity. However, in agreement with previous reports (23), we found no difference in serum IP-10 between AR patients and HCs. This suggests that the decreased CXCR3 in peripheral CD4^+^T cells may not be a responsive upregulation induced by IP-10 stimulation through a negative feedback loop. On the contrary, several studies indicate that IP-10 and MIG levels are elevated in nasal lavages taken from AR patients, suggesting their roles in chronic inflammation caused by allergic responses (18, 24). In addition to CXCR3, AR also induce MFI downregulation of CCR4 and CCR6, suggesting a reduced migratory capacity and accumulation of T cells in local tissue of AR patients. Since several reports have indicated local accumulation of T cells in nasal mucosal (25, 26), our study may provide a source of those accumulated T cells. However, we did not include T cell functional experiments and T cell samples from nasal tissue in this study, which may differ from those in peripheral blood. This limitation may impede us from drawing the conclusion. Further study will be performed to compare chemokine receptor expression and function between T cells in peripheral and local nasal tissue.

The low T cell expression of CXCR3 may influence the downstream signals triggered by crosslinking of CXCR3 and IP-10/MIG. This hypothesis is supported by a previous study, in which cells from AR patients were found to be hypo-responsive to CXCR3 ligands, MIG, IP-10, and I-TAC, promoting a diminished Th1 response compared to non-allergic controls (27). In addition, several studies have suggested that drugs inducing local and peripheral increases in IFN-γ and IP-10 are associated with a reduction of AR symptoms (28, 29). Overall, our study suggests that CD4^+^T cell CXCR3 expression is a key factor in the pathogenesis of AR, and may serve as a potential disease and/or therapeutic target.

## Data Availability Statement

The original contributions presented in the study are included in the article/supplementary material. Further inquiries can be directed to the corresponding author.

## Ethics Statement

The studies involving human participants were reviewed and approved by Ethics approval was obtained from the Ethics Committee of Shengjing hospital affiliated to CMU, and written informed consent for participation in the study was obtained from all study participants. The patients/participants provided their written informed consent to participate in this study.

## Author Contributions

XY and ZC designed experiments, analyzed data, and wrote the manuscript with contributions from all authors. XY, ZC and MW performed clinical donor recruitment, patient evaluation. XY performed all the experiments. ZC directed and supervised the study. All authors listed have made a substantial, direct, and intellectual contribution to the work and approved it for publication.

## Funding

This work was supported by Educational Commission of Liaoning Province (JC2019010), and Science and technology foundation projects of Liaoning Province (20180550027).

## Conflict of Interest

The authors declare that the research was conducted in the absence of any commercial or financial relationships that could be construed as a potential conflict of interest.

## Supplementary Material

Supplementary Figure 1 Patent chemokine receptor expression based on different allergens.

## References

[1] Zhang H Cardell LO Bjorkander J Benson M Wang H Comprehensive Profiling of Peripheral Immune Cells and Subsets in Patients with Intermittent Allergic Rhinitis Compared to Healthy Controls and After Treatment with Glucocorticoids. Inflammation (2013) 36(4):821–9. 10.1007/s10753-013-9608-0 23413042

[2] Ciprandi G Filaci G Battaglia F Fenoglio D Peripheral Th-17 cells in allergic rhinitis: New evidence. Int Immunopharmacol (2010) 10(2):226–9. 10.1016/j.intimp.2009.11.004 19925886

[3] Wang X-Q Ke X Shen Y Kang H-Y Gu Z Hu G-h Changes in circulating follicular helper T-cells in Chinese patients with allergic rhinitis. Acta Otolaryngol (2016) 136(2):199–204. 10.3109/00016489.2015.1093169 26472169

[4] Poggi A Canevali P Contatore M Ciprandi G Higher Frequencies of CD161(+) Circulating T Lymphocytes in Allergic Rhinitis Patients Compared to Healthy Donors. Int Arch Allergy Immunol (2012) 158:151–6. 10.1159/000330903 22286340

[5] Sani MM Ashari NSM Abdullah B Wong KK Musa KI Mohamud R Reduced CD4+ terminally differentiated effector memory T cells in moderate-severe. Asian Pac J Allergy Immunol (2019) 37(3):138–46. 10.12932/AP-191217-0220. 29981564

[6] Campbell JD HayGlass KT T cell chemokine receptor expression in human Th1- and Th2-associated diseases. Arch Immunol Ther Exp (Warsz) (2000) 48(6):451–6. 11197598

[7] Acosta-Rodriguez EV Rivino L Geginat J Jarrossay D Gattorno M Lanzavecchia A Surface phenotype and antigenic specificity of human interleukin 17-producing T helper memory cells. Nat Immunol (2007) 8(6):639–46. 10.1038/ni1467 17486092

[8] Brozek JL Bousquet J Agache I Agarwal A Bachert C Bosnic-Anticevich S Allergic Rhinitis and its Impact on Asthma (ARIA) guidelines-2016 revision. J Allergy Clin Immunol (2017) 140(8):950–8. 10.1016/j.jaci.2017.03.050 28602936

[9] Becattini S Latorre D Mele F Foglierini Perez M Gregorio C Cassotta A T cell immunity. Functional heterogeneity of human memory CD4^+^ T cell clones primed by pathogens or vaccines. Sci (New York NY) (2014) 347(6220):400–6. 10.1126/science.1260668 25477212

[10] Kirmaz C Bayrak P Yilmaz O Yuksel H Effects of glucan treatment on the Th1/Th2 balance in patients with allergic rhinitis: A double-blind placebo-controlled study. Eur Cytokine Netw (2005) 16:128–34. 15941684

[11] Pawankar R Mori S Ozu C Kimura S Overview on the pathomechanisms of allergic rhinitis. Asia Pac Allergy (2011) 1(3):157–67. 10.5415/apallergy.2011.1.3.157 PMC3206239 22053313

[12] Sani MM Ashari NSM Abdullah B Wong KK Musa K II Mohamud R Reduced CD4+ terminally differentiated effector memory T cells in moderate-severe house dust mites sensitized allergic rhinitis patients. Asian Pac J Allergy Immunol (2019) 37(3):138–46. 10.12932/ap-191217-0220 29981564

[13] Borish L Allergic rhinitis: Systemic inflammation and implications for management. J Allergy Clin Immunol (2003) 112(6):1021–31. 10.1016/j.jaci.2003.09.015 14657851

[14] Schmidt-Weber CB Akdis M Akdis CA TH17 cells in the big picture of immunology. J Allergy Clin Immunol (2007) 120(2):247–54. 10.1016/j.jaci.2007.06.039 17666214

[15] Lokensgard JR Hu S Sheng W vanOijen M Cox D Cheeran MCJ Robust expression of TNF-α, IL-1β, RANTES, and IP-10 by human microglial cells during nonproductive infection with herpes simplex virus. J NeuroVirol (2001) 7(3):208–19. 10.1080/13550280152403254 11517395

[16] Wong CK Lam CWK Wu AKL Ip WK Lee NLS Chan IHS Plasma inflammatory cytokines and chemokines in severe acute respiratory syndrome. Clin Exp Immunol (2004) 136(1):95–103. 10.1111/j.1365-2249.2004.02415.x 15030519 PMC1808997

[17] Velazquez-Salinas L Verdugo-Rodriguez A Rodriguez LL Borca MV The Role of Interleukin 6 During Viral Infections. Front Microbiol (2019) 10:1057. 10.3389/fmicb.2019.01057 31134045 PMC6524401

[18] Campbell JD Stinson MJ Simons FER HayGlass KT Systemic chemokine and chemokine receptor responses are divergent in allergic versus non-allergic humans. Int Immunol (2002) 14(11):1255–62. 10.1093/intimm/dxf098 12407016

[19] Okamoto T Iwata S Ohnuma K Dang NH Morimoto C Histamine H1-receptor antagonists with immunomodulating activities: potential use for modulating T helper type 1 (Th1)/Th2 cytokine imbalance and inflammatory responses in allergic diseases. Clin Exp Immunol (2009) 157(1):27–34. 10.1111/j.1365-2249.2009.03958.x 19659767 PMC2710589

[20] Sallusto F Lenig D Mackay CR Lanzavecchia A Flexible programs of chemokine receptor expression on human polarized T helper 1 and 2 lymphocytes. J Exp Med (1998) 187(6):875–83. 10.1084/jem.187.6.875 PMC2212187 9500790

[21] Pandya JM Lundell A-C Andersson K Nordström I Theander E Rudin A Blood chemokine profile in untreated early rheumatoid arthritis: CXCL10 as a disease activity marker. Arthritis Res Ther (2017) 19(1):20–0. 10.1186/s13075-017-1224-1 PMC5289001 28148302

[22] Cui L-Y Chu S-F Chen N-H The role of chemokines and chemokine receptors in multiple sclerosis. Int Immunopharmacol (2020) 83:106314. 10.1016/j.intimp.2020.106314 32197226 PMC7156228

[23] Sun J Wong B Cundall M Goncharova S Conway M Dalrymple A Immunoreactivity profile of peripheral blood mononuclear cells from patients with ragweed-induced allergic rhinitis. Clin Exp Allergy (2007) 37(6):901–8. 10.1111/j.1365-2222.2007.02723.x 17517104

[24] Mazzi V Fallahi P Allergic rhinitis and CXCR3 chemokines. Clin Ter (2017) 168(1):e54–8. 10.7417/CT.2017.1983 28240764

[25] Nishimura T Kaminuma O Saeki M Kitamura N Gotoh M Mori A Effects of anti-allergic drugs on T cell-mediated nasal hyperresponsiveness in a murine model of allergic rhinitis. Allergol Int (2018) 67:S25–31. 10.1016/j.alit.2018.05.002 29910099

[26] Kaminuma O Nishimura T Saeki M Mori A Hiroi T T Cell-Mediated Nasal Hyperresponsiveness in Allergic Rhinitis. Biol Pharm Bull (2020) 43(1):36–40. 10.1248/bpb.b18-01021 31902929

[27] Campbell JD Gangur V Simons FER HayGlass KT Allergic humans are hyporesponsive to a CXCR3 ligand-mediated Th1 immunity-promoting loop. FASEB J (2004) 18(2):329–31. 10.1096/fj.02-0908fje 14657006

[28] Tsitoura D Ambery C Price M Powley W Garthside S Biggadike K Early clinical evaluation of the intranasal TLR7 agonist GSK2245035: Use of translational biomarkers to guide dosing and confirm target engagement. Clin Pharmacol Ther (2015) 98(4):369–80. 10.1002/cpt.157 26044169

[29] Tanimoto A Ogawa Y Oki C Kimoto Y Nozawa K Amano W Pharmacological properties of JTE-052: a novel potent JAK inhibitor that suppresses various inflammatory responses in vitro and in vivo. Inflamm Res (2015) 64(1):41–51. 10.1007/s00011-014-0782-9 25387665 PMC4286029

