# Supplementary figures and images for: Reduced CD4+T Cell CXCR3 Expression in Patients With Allergic Rhinitis
Source: Front Immunol. 2020 Nov 3;11:581180. doi: 10.3389/fimmu.2020.581180 (PMC7669911; doi:10.3389/fimmu.2020.581180)

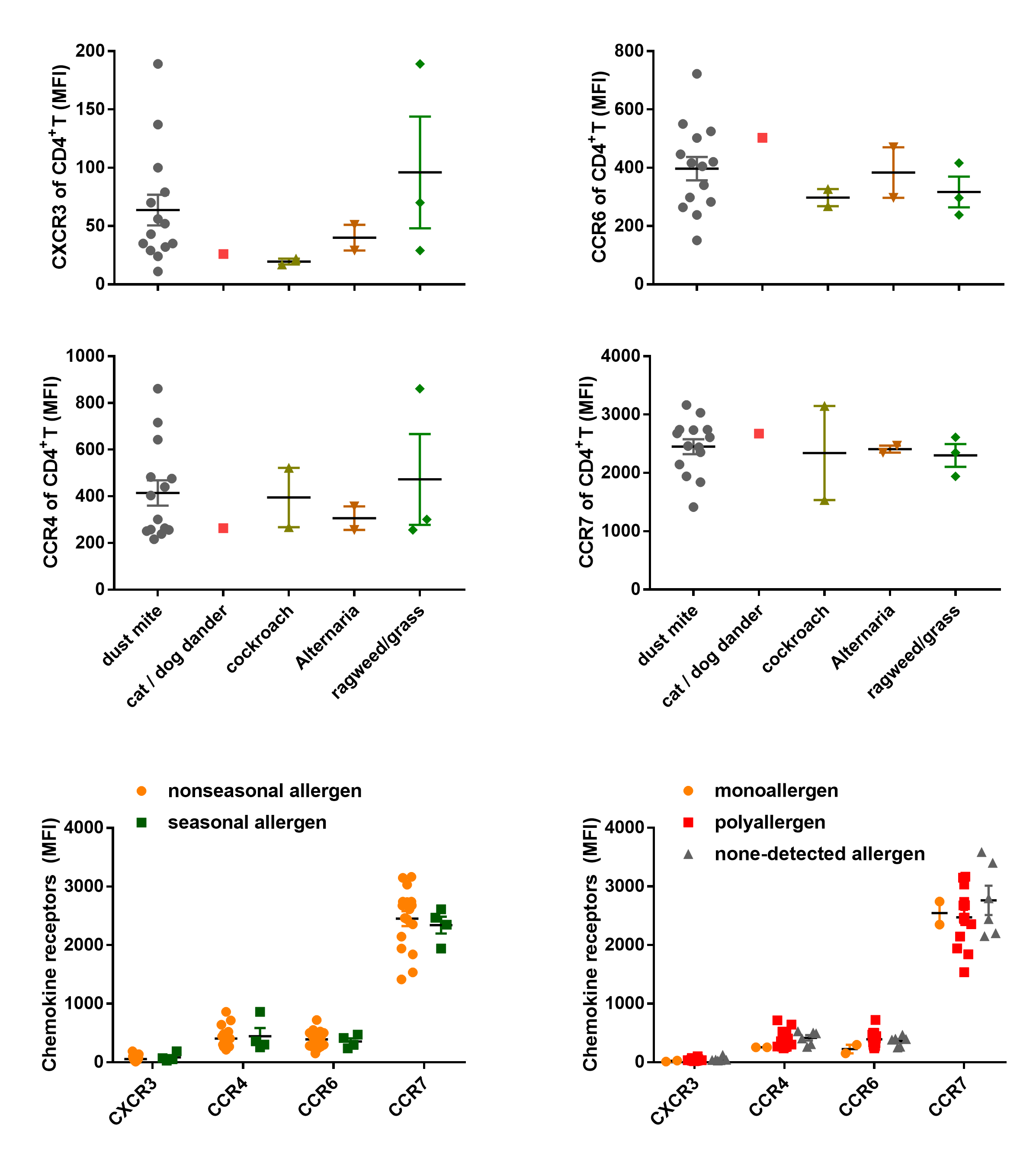

Supplement: Supplementary Figure 1 — Patent chemokine receptor expression based on different allergens. [file Image_1.tif]
